# Occurrence of *Salmonella* in the Cattle Production in France

**DOI:** 10.3390/microorganisms9040872

**Published:** 2021-04-17

**Authors:** Laetitia Bonifait, Amandine Thépault, Louise Baugé, Sandra Rouxel, Françoise Le Gall, Marianne Chemaly

**Affiliations:** Unit of Hygiene and Quality of Poultry and Pork Products, French Agency for Food, Environmental and Occupational Health and Safety (ANSES), Laboratory of Ploufragan-Plouzané-Niort Site BP 53, 22440 Ploufragan, France; amandine.thepault@anses.fr (A.T.); louise.bauge@anses.fr (L.B.); sandra.rouxel@anses.fr (S.R.); francoise22200@gmail.com (F.L.G.); marianne.chemaly@anses.fr (M.C.)

**Keywords:** *Salmonella*, cattle production, whole genome sequencing, France

## Abstract

*Salmonella* is among the most common foodborne pathogens worldwide, and can lead to acute gastroenteritis. Along with poultry, cattle production is recognized as an important source of human infection. *Salmonella* transmission from cattle to humans can occur through the environment, or through close contact with sick animals or their derived products. This study aimed to investigate the intestinal carriage of *Salmonella* spp. within French cattle production. A total of 959 cattle intestinal samples, from one of the largest French slaughterhouses, were analyzed. Isolated strains were genotyped by pulsed field gel electrophoresis (PFGE), and a sub-selection was taken by whole genome sequencing (WGS). Twenty-nine samples were positive for *Salmonella* spp., yielding an estimated prevalence of 3% in cattle production. Eight different *Salmonella* serotypes were found: Montevideo was the most prevalent (34%), followed by Mbandaka (24%) and Anatum (14%). PFGE genotyping allowed the clustering of *Salmonella* isolates according to their serotype. Within the clusters, some isolates presented 100% similarity. To investigate potential epidemiological links between them, WGS and core genome multilocus sequence typing (cgMLST) were used, revealing identical profiles between isolates originating from different areas and/or different animal breeds. This investigation provides new insights on *Salmonella* serotype epidemiology in cattle production in France.

## 1. Introduction

Salmonellosis is an important public health problem among the common bacterial foodborne zoonoses, which contributes to economic losses in both developed and developing countries. Worldwide, it remains the second most commonly reported gastrointestinal infection in humans after campylobacteriosis, and is a significant cause of foodborne outbreaks [1]. Nontyphoidal *Salmonella* spp. are responsible for the highest annual burden and the largest number of deaths both globally and in the European Region [2]. Worldwide, *Salmonella* spp. were estimated to be responsible for 78 million cases of illness annually, 230,000 deaths and 4 million disability-adjusted life years (DALYs) per year [3,4]. In 2019, 87,923 confirmed cases of salmonellosis in humans were reported in Europe. Nontyphoidal *Salmonella* are bacterial enteric pathogens associated with animal food reservoirs, predominantly transmitted to humans by contaminated food and water. Among these, *Salmonella enterica* subspecies *enterica* is the most important subspecies affecting humans and domestic animals [5]. 

*Salmonella* have been widely reported in cattle [6], and dairy cows are known to be reservoirs of *Salmonella* enterica. Usually, human salmonellosis is attributed to the consumption of contaminated poultry meat and eggs, as well as dairy and beef products [7]. This presence of *Salmonella* in cattle may result in the contamination of milk or meat in the farm environment (animals, rodents, wild animals, insects, water, etc.), which could lead to direct and indirect infection of people and animals [8,9]. Infected animals may develop symptoms or shed *Salmonella* in their feces without showing any clinical signs of disease. Clinical manifestations of bovine salmonellosis include mainly apathy, hyperthermia, difficulty breathing, panting, pallor of the mucous membranes, and forthe most commonly reported manifestation, abortion [10,11]. The risk associated with asymptomatic carriage is that cattle may introduce this organism into an abattoir, which can represent a significant food safety hazard of cross-contamination during food processing [8]. Asymptomatic carriage may not impact dairy production, but herds with high *S. enterica* burden may pose an increased public health risk through contamination of the production environment and possibly milk and meat [7]. Importantly, *Salmonella* can persist in the environment for several months and be associated with wildlife, which is a reservoir for *Salmonella* and may serve as a source of contamination.

The overall prevalence in cattle has been reported by the EFSA and ECDC to be 3.34% in the European Union (the prevalence of positive samples at the slaughterhouse was 7.76%) [1]. Moreover, prevalence estimates vary from 2% in Europe to 16% in North America [12,13,14]. Studies have reported 5% in Africa, 4% in Turkey and 2% in Iran [15,16,17]. Fossler et al. showed the presence of *Salmonella* spp. in more than 90% of the environment on dairy farms in the United States [9]. 

*Salmonella* Dublin, *Salmonella* Newport, and *Salmonella* Typhimurium are generally associated with salmonellosis in calves and adult cows, causing mild to severe illness. Moreover, asymptomatic carriage and fecal shedding of *Salmonella* serotypes such as *Salmonella* Cerro, *Salmonella* Kentucky, *Salmonella* Mbandaka, and *Salmonella* Montevideo have been well documented in dairy animals [10,18]. 

The objectives of this study were to investigate the occurrence of *Salmonella* in cattle production in France, serotype distribution, genetic diversity of *Salmonella* isolates and possible epidemiological links among the isolates, in order to better understand *Salmonella* epidemiology, and potentially to achieve a better control of *Salmonella* in cattle production.

## 2. Materials and Methods

### 2.1. Sampling Plan

Bovine intestinal content samples were taken from one of the largest slaughterhouses in France. A total of 959 intestinal samples were randomly collected from the slaughter line at the evisceration step, over a period of 6 months with a frequency of 50 samples per week. The 959 samples originated from 282 farms distributed across 32 French areas, representative of cattle production in the country. Among the samples, 476 were from calves (less than 8 months old) and 483 were from adult cattle (Table 1). Samples were transported to the laboratory in isotherm bags and stored at +4 °C until analysis.

### 2.2. Salmonella Isolation and Quantification

Samples of 25 g were analyzed for *Salmonella* detection according to the NF EN ISO 6579 and NF U 47-100 standards [19,20]. Samples were homogenized in a 1:10 sample:broth ratio in a Pulsifier^®^ (Microgen Bioproducts, Surrey, United-Kingdom) with 225 mL of buffered peptone water (Biomérieux, Craponne, France) for pre-enrichment. After incubation, for 18 ± 2 h at 37 °C, 0.1 mL was transferred to modified semi-solid Rappaport-Vassiliadis) (MSRV) medium (Biokar, Beauvais, France) and incubated for 24 h, then 48 h at 41.5 °C. One milliliter of pre-enrichment broth was also inoculated to 10 mL of Muller Kauffmann tetrathionate broth (MKTTn) (Biokar, France) and incubated for 24 h at 37 °C. Cultures obtained from MSRV were inoculated on xylose lysine deoxycholate (XLD) agar (Biokar, France) and Rapid’*Salmonella* (R’S) agar (BioRad, Paris, France), and cultures obtained from MKTTn on Xylose Lysine Tergitol 4 (XLT4) agar (Biokar, France). Colonies of presumptive *Salmonella* were subcultured and their identity was confirmed by biochemical assays for glucose fermentation, lactose oxidation, gas, H2S production (triple sugar iron (TSI) agar, Biokar, France), lack of galactosidase (ONPG), and presence of decarboxylase (L-Lysine). All *Salmonella* isolates were confirmed by serotyping according to the Kauffmann–White scheme using slide agglutination tests [21].

In parallel, *Salmonella* enumerations were carried out with the miniaturized most probable number (mMPN) technique, according to the XP CEN ISO/TS 6579-2 standard [22]. 

### 2.3. Genotyping and Genotypes Comparisons

#### 2.3.1. Pulsed-Field Gel Electrophoresis

Pulsed-field gel electrophoresis (PFGE) of isolates of *Salmonella* was performed using the *Xba*I and *Bln*I restriction enzymes following the procedure developed by the US Center for Disease Control (CDC) [23] and as previously described by Kerouanton et al. [24]. PFGE fingerprints were analyzed (fragment size estimation) using BioNumerics v.7.5 software (Applied Maths, Belgium). Similarities between isolate fingerprints were calculated using the Dice index, with a maximum tolerance of 1%, and a dendrogram was built using the unweighted pair group method with arithmetic mean (UPGMA algorithm) [24]. 

#### 2.3.2. Whole Genome Sequencing

Whole genome sequencing (WGS) was performed on isolates of *Salmonella* serotypes collected in at least 2 animals to investigate potential epidemiological links between them. The WGS was performed following DNA extraction from the strains. DNA was extracted from one-day single colony cultures with a QIAamp DNA Mini Kit (QIAGEN, Villebon-sur-Yvette, France), according to the manufacturer’s instructions, and quantified using a Qubit 2.0 fluorometer and a Qubit dsDNA (double-stranded DNA) HS (high-sensitivity) assay kit (Thermo Fisher Scientific, Villebon-sur-Yvette, France). WGS was performed using Illumina technology at the technical center “Institut du Cerveau et de la Moelle épinière”, Pitié-Salpêtrière Hospital, Paris (www.icm-institute.org, accessed on 16 April 2021). Libraries were prepared using a Nextera XT DNA Library Preparation Kit and Nextera XT Index Kit (Illumina, Paris, France). Samples were then sequenced with a NextSeq 500 machine using a NextSeq 500 Mid Output Kit v2 (300 cycles), (Illumina, France). Paired-end raw reads were deposited on the EnteroBase database platform for *Salmonella* (http://enterobase.warwick.ac.uk/, accessed on 16 April 2021), and were automatically de novo assembled using SPAdes [25] once the sequences were uploaded. 

Genomic comparison of the isolated strains was carried out using the cgMLST scheme with 3002 genes included, available on EnteroBase [26]. Similar but nonidentical strains (strains showing different core genome Sequence Type (cgST)) were identified in EnteroBase by using the hierarchical clustering method (HierCC) that allows for grouping of strains into hierarchical clusters (HCs) that can differ up to a specified and fixed number of cgMLST alleles. This number is indicated by the suffix following “HC” (e.g., HC5 for 5 cgMLST allelic differences). To assess the genetic relationship between strains of the same serotype and the population structure of *Salmonella* isolates, a neighbor-joining tree was created from cgMLST allelic differences in EnteroBase using GrapeTree [27] and the RapidNJ algorithm [28]. The assembly sequences are publicly available from EnteroBase; their accession numbers (barcodes) are listed in Table 3.

### 2.4. Statistical Analysis

The statistical relationship between the prevalence of *Salmonella* in calves or adult cattle was analyzed by Chi-Square test. Meanwhile, the statistical relationship between positive dairy and beef cows was analyzed by Fisher’s exact test. *p*-Value ≤ 0.05 was considered as statistically significant.

## 3. Results

### 3.1. Estimation of Salmonella Prevalence and Quantification

**Figure 1:**
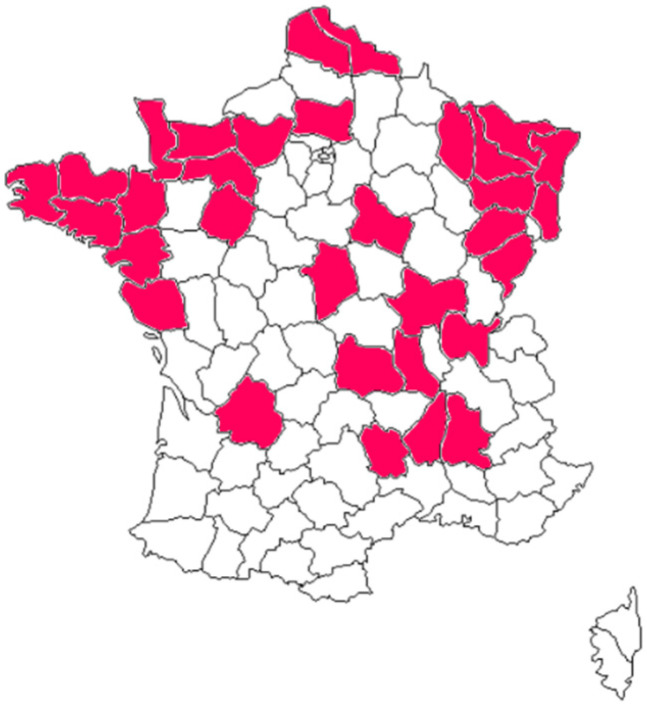
Geographical distribution of the sampled cattle from breeding areas throughout the country.

In all, 29 out of 959 samples were positive for *Salmonella* spp. (Table 2), suggesting a 3% prevalence in cattle production in France. Intestinal contents were taken from animals originating from 32 different breeding areas (Figure 1). *Salmonella* strains were isolated from animals from 14 of these areas (Table 2). 

Among the positive samples, 55% (16/29) and 45% (13/29) of isolates were from intestinal contents of calves and adults, respectively, which represents a prevalence of 3.4% (16/476) for calves and 2.7% (13/483) for adult cattle. No statistical difference was found for *Salmonella* carriage according to animal age (*p* > 0.05). *Salmonella* was detected among several cattle breeds (Table 2) but there was no statistical difference between *Salmonella* positive dairy cows (3.7%; 20/541) and beef cows (2.2%; 9/413) (*p* > 0.05).

Among all the positive samples, 55% (16/29) were quantified using the mMPN method. The range of *Salmonella* concentrations found in all samples varied from 2 CFU/g to >710 CFU/g (Table 2). More precisely, 27% of samples (8/29) had a load range between 2 and 100 CFU/g, 14% (4/29) between 100 and 700 CFU/g, and 14% (4/29) greater than 710 CFU/g.

### 3.2. Serotype

Eight serotypes of *Salmonella* were identified among the 29 positive samples. *Salmonella* Montevideo was the most prevalent serotype (34%; 10/29), followed by *Salmonella* Mbandaka (24%; 7/29) and *Salmonella* Anatum (14%; 4/29). 

**Figure 2:**
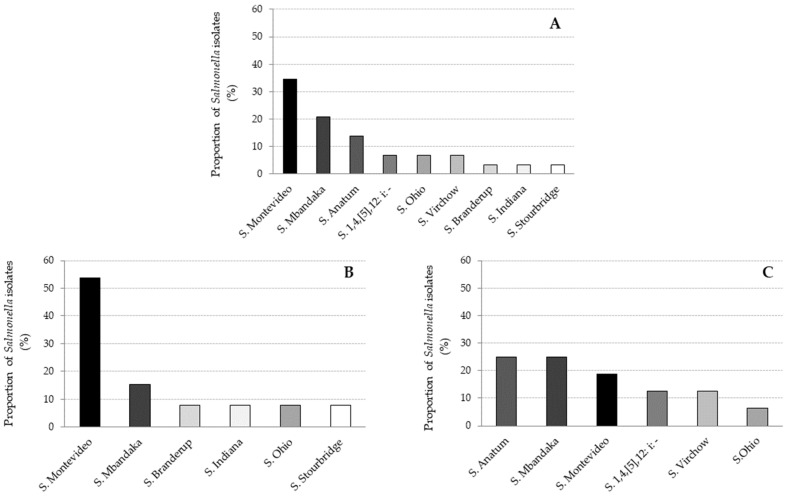
Main *Salmonella* serotypes found in the intestinal contents from all sampled animals (**A**), adult cattle (**B**) and calves (**C**). Each color represents one serotype of *Salmonella*.

The other serotypes, *Salmonella* Stourbridge, *Salmonella* Ohio, *Salmonella* Indiana, *Salmonella* Virchow and *Salmonella* Typhimurium monophasic variant (1,4,[5],12: i: −), were more rarely isolated (<10%) (Figure 2A). The distribution of *Salmonella* serotypes was slightly different between adult cattle (Figure 2B) and calves (Figure 2C). In adults, *S*. Montevideo was the most prevalent serotype, while in calves, *S*. Anatum and *S*. Mbandaka predominated. Moreover, *S*. Anatum was only found in calves.

### 3.3. Genotyping

**Figure 3:**
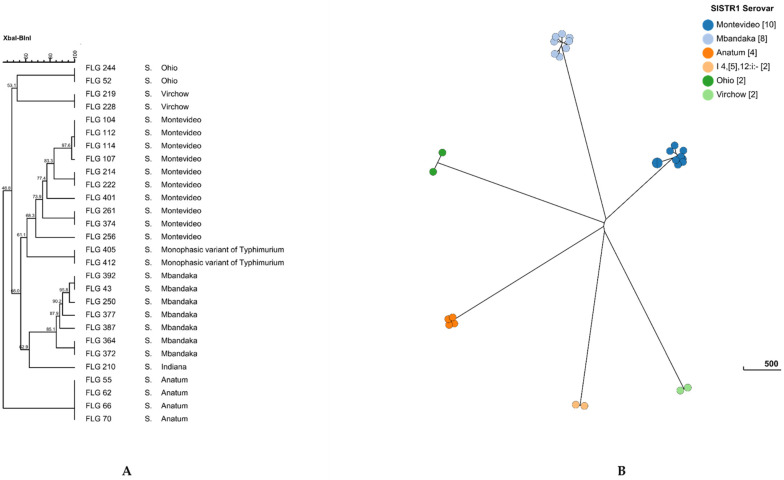
Dendrogram of pulsed-field gel electrophoresis (PFGE) cluster analysis using the *Xba*I and *Bln*I restriction enzymes (**A**) and cgMLST Grape Tree (**B**) of selected isolates of *Salmonella*.

As expected, pulsed-field genotyping clustered *Salmonella* isolates according to their serotypes (Figure 3A). The *S*. Stourbridge isolate was not genotyped by PFGE since no growth was observed during subculture. Within the clusters, some isolates, such as *S*. Anatum or *S*. Virchow, presented 100% similarity using two restriction enzymes (XbaI and BlnI), while *S*. Montevideo and *S*. Mbandaka showed higher diversity between the isolates, since they showed from 67% to 83% similarity (Figure 3A). Interestingly, clustered isolates of *S*. Anatum and *S*. Ohio originated from different birth areas and/or from different animal breeds, independently of the serotype.

Among the 29 collected isolates of *Salmonella*, 27 were sequenced; *S*. Indiana and *S*. Stourbridge isolates were excluded for the WGS, since these serotypes were isolated from only one animal and no link can be drawn with other animals. The genomes of *Salmonella* strains were compared using the core-genome MLST approach (cgMLST scheme available on EnteroBase, https://enterobase.warwick.ac.uk, accessed on 16 April 2021). The 27 sequenced strains harbored a unique cgST profile (Table 3), indicating that they are all different and showed at least one cgMLST allelic variation between one another. As previously described in this study using PFGE (Figure 3A), with WGS (prediction serotype, SISTR1 and using the Sequence Type MLST), the strains belonging to the same serotype clustered together (Table 3 and Figure 3B). This clustering of the strains according to their serotype was also illustrated at the HC200 level, since each strain of the same serotype harbored the same HC200|cluster (Table 3). Here, we considered that the isolates clustered in the same node on the neighbor-joining tree (Figure 3B) and belonging to the same HC5|cluster (up to five allelic variations between strains of the same HC5|cluster) are highly probably epidemiologically linked.

Within *S*. Anatum, three isolates (S16LNR-FLG55, S16LNR-FLG62 and S16LNR-FLG70) were grouped in the same HC5|cluster, HC5|215760. Among these isolates, two (S16LNR-FLG55 and S16LNR-FLG70) harbored the same HC2|cluster, indicating a maximum of two allelic variations. Interestingly, within the three clustered isolates, two were originated from animals bred in the same geographic area, indicating a high probability of an epidemiological link between these isolates. 

For *S*. Typhimurium monophasic variant isolates (S16LNR-FLG405 and S16LNR-FLG412), *S*. Mbandaka isolates (S16LNR-FLG372 and S16LNR-FLG364), and *S*. Montevideo isolates (S16LNR-FLG214 and S16LNR-FLG222), although the PFGE genotypes presented 100% similarity (Figure 3), WGS and cgMLST results differentiated the strains up to the HC200 level (a maximum of 200 cgMLST allelic variations). Therefore, it did not allow for inferring any epidemiological links between these isolates, despite a common breeding area for *S*. Typhimurium monophasic variant and *S.* Mbandaka isolates. 

Regarding *S*. Virchow isolates, WGS and cgMLST confirmed the genotyping results found using PFGE. The two strains clearly grouped in the same HC10|cluster (Table 3), suggesting a possible epidemiological link between the strains isolated from animals bred in the same geographic area (Table 2).

Genotyping by PFGE showed two other clusters of *S*. Montevideo with about 100% similarity, the first was composed of four strains (S16LNR-FLG104, S16LNR-FLG107, S16LNR-FLG112 and S16LNR-FLG114) and the second of two strains of *S*. Montevideo (S16LNR-FLG261 and S16LNR-FLG374). Within the first PFGE cluster, two strains belonged to the same HC2 cluster (S16LNR-FLG112 and S16LNR-FLG114), and three (S16LNR-FLG107, S16LNR-FLG112 and S16LNR-FLG114) grouped in the same HC10|cluster (Table 3). The fourth isolate, S16LNR-FLG104, belonged to different HC10|cluster, indicating that it differs from the others three strains by at least 10 allelic variations. Regarding the second PFGE cluster within *S*. Montevideo isolates, two (S16LNR-FLG261 and S16LNR-FLG374) harbored different cgST profiles; however, they belonged to the same HC0|cluster, HC0|215773. These cgMLST results indicate that all alleles of the core genes present in the strains are identical (same HC0|cluster), but the strains differed in the presence/absence of some core genes (cgST different). Here, an epidemiological link could be probable, despite different birth and breeding areas. 

## 4. Discussion

Foodborne illnesses are a major public health concern and result in considerable economic burden. *Salmonella* has the ability to adapt to a variety of animal hosts, and to humans, and can be transmitted through contaminated food such as eggs, meat, raw vegetables, or through water [29]. More specifically, bovine salmonellosis is responsible for public and animal health problems and serious economic losses due to high mortality, and is often caused by *Salmonella* Dublin [11,30]. 

The prevalence of *Salmonella* in cattle found in this study (3%) is consistent with the data found in the literature for Europe, where asymptomatic carriage of *Salmonella* in cattle is generally less than 5% based on fecal samples [8,31]. It is possible that the incidence of salmonellosis was underestimated, since fecal samples are not necessarily the most sensitive source to detect the presence of *Salmonella*, and some positive cattle may have been missed by the detection method used [8,17,32]. In addition, prevalence may be underestimated because the animals sampled at the slaughterhouse are healthy, which does not take into account symptomatic cattle carrying *Salmonella*. The prevalence of *Salmonella* in apparently healthy cattle is of significant concern to public health [17], especially that they are able to carry more than 700 CFU per gram of *Salmonella*, as shown in this study, and thus may cross-contaminate the meat products during processing. 

In this investigation, neither the age of animals (calf: 3.4%; adult: 2.7%) nor the type of cattle (dairy cow: 3.7%; beef cow: 2.2%) were discriminating factors for *Salmonella* carriage, despite some studies showing the opposite [32]. It was also reported that the prevalence of *Salmonella* in animal and environmental samples varied among seasons, with an increase in the presence of *Salmonella* on dairy farms when the seasonal temperature increased [33]. This intensification was even observed during the period from August to October in an Irish slaughterhouse [8].

Within the 29 of 959 samples collected that were positive for *Salmonella* enterica, eight different serotypes were isolated. *Salmonella* Montevideo was identified as the most prevalent, followed by *S.* Mbandaka and *S.* Anatum. Several studies have highlighted the presence of theses serotypes, and particularly *S.* Montevideo as the predominant serotype in cattle [12,13,14,34]. Although *S.* Montevideo and *S.* Dublin are the most frequently reported serotypes in North America and Europe, no *S.* Dublin was found in this investigation [8,31,35,36]. These results can be explained by the possible presence of symptoms in animals infected with *S.* Dublin, which would have resulted in the eviction of these animals from slaughter. Nevertheless, other serotypes such as *S.* Virchow and *S.* Typhimurium monophasic variant, commonly found in cattle, were also isolated [31]. These results also show variability in the serotypes of *Salmonella* in cattle production between calves and adult cattle.

The geographic distribution of breeding areas did not influence *Salmonella* carriage. However, PFGE and WGS genotyping highlighted genetic similarities, suggesting potential epidemiological links or cross-contaminations between animals. The available metadata were not sufficiently detailed to identify the pathways of contamination of the cattle by *Salmonella*. Nevertheless, this investigation made it possible to assess whether the contamination occurred at the place of birth of the animals, or at the breeding farm, and to put forward the hypothesis of possible contamination during transport or at the slaughterhouse (when no links between the strains were found). As an example, certain strains of *S.* Anatum presented strong evidence of an epidemiological link between isolates originating from different areas of birth and breeding. Proximity between the areas might suggest possible circulation of the same *S.* Anatum strain throughout these areas of France. However, in the case of *S.* Virchow, contamination appeared to have taken place on the farm, at the breeding step, since the areas of birth of the calves were different. Linking exhaustive epidemiological data and WGS genotyping would allow the establishment of reliable epidemiological links, which are needed to understand *Salmonella* contamination in cattle production. 

## 5. Conclusions

This investigation made it possible, for the first time, to evaluate the intestinal carriage of *Salmonella* by cattle in France and to identify *S.* Montevideo as the most common serotype. The use of WGS to genotype strains enabled the identification of possible epidemiological links among the *Salmonella* strains in cattle. The knowledge gained in this investigation about the prevalence and diversity of *Salmonella* serotypes will help to improve understanding about the dissemination of *Salmonella* in French cattle production, to adapt the current control measures, and to prevent public health problems.

## Figures and Tables

**Table 1:** Number of cattle intestinal samples studied in this work, distributed by the month of sampling, age of cattle (cows and calves) and productive aptitude (dairy and beef).

Month of Sampling	Number of Samples Collected						
	Cows			Calves			
	Dairy	Beef	% vs. Total Cows’ Samples	Dairy	Beef	Mixed/ Unknown *	% vs. Total Calves’ Samples
June	30	11	8.5%	46	2		10.1%
July	46	54	20.7%	43	56	1	21.0%
August	51	49	20.7%	84	16		21.0%
September	33	67	20.7%	41	44		17.9%
October	21	69	18.6%	39	11		10.5%
November	23	14	7.7%	55	3		12.2%
December	9	6	3.1%	20	11	1/3	7.4%
Total number of samples	213	270	483	328	143	5	476

* The mention “mixed” indicates a mixed breed, the mention “unknown” indicates the absence of information about the breed of the animal.

**Table 2:** List of the 29 *Salmonella* positive samples in this study.

Sample Name	Cattle Age Category	Breed Type	Cattle Birth Area	Cattle Breeding Area	*Salmonella* Serotype Isolated	*Salmonella* Enumeration CFU/g
S16LNR-FLG405	Calf	Dairy	E	E	*S.* 1,4,[5],12: i: −	ND
S16LNR-FLG412	Calf	Dairy	C	E	*S.* 1,4,[5],12: i: −	22
S16LNR-FLG55	Calf	Dairy	O	H	*S.* Anatum	8
S16LNR-FLG62	Calf	Dairy	C	C	*S.* Anatum	380
S16LNR-FLG66	Calf	Dairy	F	E	*S.* Anatum	2
S16LNR-FLG70	Calf	Dairy	C	C	*S.* Anatum	220
S16LNR-FLG210	Adult	Dairy	B	B	*S.* Indiana	6
S16LNR-FLG43	Adult	Beef	K	K	*S.* Mbandaka	>710
S16LNR-FLG250	Adult	Beef	L	H	*S.* Mbandaka	ND
S16LNR-FLG364	Calf	Dairy	N	F	*S.* Mbandaka	2
S16LNR-FLG372	Calf	Dairy	S	F	*S.* Mbandaka	ND
S16LNR-FLG377	Adult	Beef	M	M	*S.* Mbandaka	ND
S16LNR-FLG387	Calf	Dairy	O	D	*S.* Mbandaka	ND
S16LNR-FLG392	Calf	Dairy	T	D	*S.* Mbandaka	ND
S16LNR-FLG104	Adult	Dairy	C	C	*S.* Montevideo	>710
S16LNR-FLG107	Adult	Beef	G	G	*S.* Montevideo	>710
S16LNR-FLG112	Adult	Dairy	M	M	*S.* Montevideo	220
S16LNR-FLG114	Adult	Dairy	N	N	*S.* Montevideo	170
S16LNR-FLG214	Calf	Dairy	P	A	*S.* Montevideo	ND
S16LNR-FLG222	Calf	Dairy	Q	A	*S.* Montevideo	19
S16LNR-FLG256	Adult	Beef	N	N	*S.* Montevideo	ND
S16LNR-FLG261	Adult	Beef	I	I	*S.* Montevideo	8
S16LNR-FLG374	Calf	Dairy	N	F	*S.* Montevideo	ND
S16LNR-FLG401	Adult	Dairy	F	F	*S.* Montevideo	>710
S16LNR-FLG52	Calf	Beef	E	E	*S.* Ohio	ND
S16LNR-FLG244	Adult	Beef	L	L	*S.* Ohio	8
S16LNR-FLG05	Adult	Dairy	J	J	*S.* Stourbridge	ND
S16LNR-FLG219	Calf	Beef	R	A	*S.* Virchow	ND
S16LNR-FLG228	Calf	Dairy	P	A	*S.* Virchow	ND

ND: Non detectable (under the detection limit of the method). Cattle birth and breading areas were anonymized; each letter was randomly attributed to a specific area. If animals were born and bred in the same French area, letters of birth and breeding areas were identical. In the same way, if several animals harbored the same letter as birth and/or breeding area, it means that the animals originated from the same area of birth and/or breeding.

**Table 3:** Twenty-seven *Salmonella* strains sequenced in this study, serotypes, sequence type (ST) and core genome MLST (cg MLST) profiles.

Strain Name	Genome Accession Number (BarCode) in EnteroBase	SISTR1 Serovar				Sequence Type (ST) MLST	Sequence Type (ST) cgMLST					
		Serotype	Serogroup	H1	H2	Achtman Scheme	Core Genome Sequence Type (cgST)	HC0	HC2	HC5	HC10	HC200
S16LNR-FLG405	SAL_BB2134AA	4: i: −	B	i	−	34	215775	215775	215775	215775	2	2
S16LNR-FLG412	SAL_BB2133AA	4: i: −	B	i	−	34	215772	215772	215772	215772	215772	2
S16LNR-FLG55	SAL_BB2119AA	Anatum	E1	e,h	1,6	64	215763	215763	215763	215760	215760	5
S16LNR-FLG62	SAL_BB2117AA	Anatum	E1	e,h	1,6	64	215760	215760	215760	215760	215760	5
S16LNR-FLG66	SAL_BB2118AA	Anatum	E1	e,h	1,6	64	215761	215761	215761	215761	215761	5
S16LNR-FLG70	SAL_BB2121AA	Anatum	E1	e,h	1,6	64	215770	215770	215763	215760	215760	5
S16LNR-FLG43	SAL_BB2111AA	Mbandaka	C1	z10	e,n,z15	413	215755	215755	215755	215755	215755	4
S16LNR-FLG250	SAL_ZA0098AA	Mbandaka	C1	z10	e,n,z15	413	199394	199394	199394	199394	199394	4
S16LNR-FLG364	SAL_ZA0099AA	Mbandaka	C1	z10	e,n,z15	413	199395	199395	199395	199395	199395	4
S16LNR-FLG372	SAL_ZA0100AA	Mbandaka	C1	z10	e,n,z15	413	199398	199398	199398	199398	199398	4
S16LNR-FLG377	SAL_ZA0101AA	Mbandaka	C1	z10	e,n,z15	413	199396	199396	199396	199396	199396	4
S16LNR-FLG387	SAL_ZA0105AA	Mbandaka	C1	z10	e,n,z15	413	199401	199401	199401	199401	199401	4
S16LNR-FLG392	SAL_ZA0102AA	Mbandaka	C1	z10	e,n,z15	413	199399	199399	199399	199399	199399	4
S16LNR-FLG104	SAL_BB2120AA	Montevideo	C1	g,m,[p],s	−	39	215759	215759	215759	215759	215759	16
S16LNR-FLG107	SAL_BB2122AA	Montevideo	C1	g,m,[p],s	−	39	215766	215766	215766	215766	215766	16
S16LNR-FLG112	SAL_BB2124AA	Montevideo	C1	g,m,[p],s	−	39	239489	239489	239489	239489	215766	16
S16LNR-FLG114	SAL_BB2125AA	Montevideo	C1	g,m,[p],s	−	39	239490	239490	239489	239489	215766	16
S16LNR-FLG214	SAL_BB2123AA	Montevideo	C1	g,m,[p],s	−	39	215764	215764	215764	215764	215764	16
S16LNR-FLG222	SAL_BB2131AA	Montevideo	C1	g,m,[p],s	−	39	215771	215771	215771	215771	215771	16
S16LNR-FLG256	SAL_BB2130AA	Montevideo	C1	g,m,[p],s	−	39	215769	215769	215769	215769	215769	16
S16LNR-FLG261	SAL_BB2129AA	Montevideo	C1	g,m,[p],s	−	39	239491	215773	215773	215773	215773	16
S16LNR-FLG374	SAL_BB2135AA	Montevideo	C1	g,m,[p],s	−	39	215773	215773	215773	215773	215773	16
S16LNR-FLG401	SAL_BB2132AA	Montevideo	C1	g,m,[p],s	−	39	215774	215774	215774	215774	215774	16
S16LNR-FLG244	SAL_BB2128AA	Ohio	C1	b	l,w	72	215767	215767	215767	215767	215767	621
S16LNR-FLG052	SAL_BB2115AA	Ohio	C1	b	l,w	72	239488	239488	239488	239488	239488	621
S16LNR-FLG219	SAL_BB2127AA	Virchow	C1	r	1,2	9	215768	215768	215768	215768	215765	715
S16LNR-FLG228	SAL_BB2126AA	Virchow	C1	r	1,2	9	215765	215765	215765	215765	215765	715

**HC**: Hierarchical clustering.

## Data Availability

Genome assemblies of *Salmonella* isolates sequenced in this study are publicly available from the *Salmonella* database on EnteroBase platform (http://enterobase.warwick.ac.uk/, accessed on 16 April 2021), their accession numbers (barcode) are listed in the paper.
